# The SNMMI and EANM Procedural Guidelines for Diuresis Renography in Infants and Children

**DOI:** 10.2967/jnumed.118.215921

**Published:** 2018-10

**Authors:** Massoud Majd, Zvi Bar-Sever, Ana Isabel Santos, Diego De Palma

**Affiliations:** 1 SNMMI Pediatric Imaging Council, Children’s National Medical Center, Washington, DC; 2 EANM Paediatric Committee, Department of Nuclear Medicine, Schneider Children’s Medical Center, Petach Tikva, Israel; 3 EANM Paediatric Committee, Nuclear Medicine Service, Hospital Garcia de Orta, Almada, Portugal; and; 4 EANM Paediatric Committee, Nuclear Medicine Unit, “Circolo” Hospital, Varese, Italy

## PREAMBLE

The Society of Nuclear Medicine and Molecular Imaging (SNMMI) is an international scientific and professional organization founded in 1954 to promote the science, technology, and practical application of nuclear medicine. Its 18,000 members are physicians, technologists, and scientists specializing in the research and practice of nuclear medicine. In addition to publishing journals, newsletters, and books, the SNMMI also sponsors international meetings and workshops designed to increase the competencies of nuclear medicine practitioners and to promote new advances in the science of nuclear medicine. The European Association of Nuclear Medicine (EANM) equally is an international scientific and professional organization promoting the nuclear medicine with about 3,500 members specializing in the research and practice of nuclear medicine. The EANM was founded in 1987.

The SNMMI/EANM will periodically define new procedural recommendations for nuclear medicine practice to help advance the science of nuclear medicine and to improve the quality of service to patients. Existing practice guidelines will be reviewed for revision or renewal, as appropriate, on their fifth anniversary or sooner, if indicated.

Each procedural recommendation, representing a policy statement by the SNMMI/EANM, has undergone a thorough consensus process in which it has been subjected to extensive review. The SNMMI/EANM recognizes that the safe and effective use of diagnostic nuclear medicine imaging requires specific training, skills, and techniques, as described in each document.

These procedural recommendations are an educational tool designed to assist practitioners in providing appropriate care for patients. They are not inflexible rules or requirements of practice and are not intended, nor should they be used, to establish a legal standard of care. For these reasons and those set forth below, the SNMMI and the EANM caution against the use of these procedural recommendations in litigation in which the clinical decisions of a practitioner are called into question.

The ultimate judgment regarding the propriety of any specific procedure or course of action must be made by the physician in light of all the circumstances presented. Thus, an approach that differs from the procedural recommendations, standing alone, does not necessarily imply that the approach was below the standard of care. To the contrary, a conscientious practitioner may responsibly adopt a course of action different from that set forth in the procedural recommendations when, in the reasonable judgment of the practitioner, such course of action is indicated by the condition of the patient, limitations of available resources, or advances in knowledge or technology subsequent to publication of the procedural recommendations.

The practice of medicine involves not only the science, but also the art of dealing with the prevention, diagnosis, alleviation, and treatment of disease. The variety and complexity of human conditions make it impossible to always reach the most appropriate diagnosis or to predict with certainty a particular response to treatment. Therefore, it should be recognized that adherence to these procedural recommendations will not ensure an accurate diagnosis or a successful outcome. All that should be expected is that the practitioner will follow a reasonable course of action based on current knowledge, available resources, and the needs of the patient to deliver effective and safe medical care. The sole purpose of these procedural recommendations is to assist practitioners in achieving this objective.

## I. INTRODUCTION

With the increasing use of sonography, dilatation of the pelvicalyceal system (hydronephrosis, HN) or ureter (hydroureteronephrosis, HUN) is the most common abnormality of the urinary tract detected in utero or after birth. Common causes of congenital HN and HUN include vesicoureteric reflux (VUR), ureteropelvic junction (UPJ) stenosis, ureterovesical junction (UVJ) stenosis, posterior urethral valve (PUV), ectopic ureter with or without duplication, ectopic ureterocele, and primary nonobstructive dilatation. Some of the affected children present with urinary tract infection or an abdominal mass, but most of them remain asymptomatic for a long time. The natural course of HN/HUN in infants and children is variable. In some patients, HN/HUN improves or resolves completely, but in others, it remains stable or gradually gets worse and, depending on the underlying cause and without timely surgical intervention, may result in silent loss of renal function or recurrent episodes of abdominal/flank pain. Therefore, the aim for imaging evaluation of these infants and children should be to identify the kidneys that are at risk for loss of function and prevent loss of function by timely surgical intervention.

Conventional dynamic renal scintigraphy provides information about the function of the affected kidney and supplements the ultrasound findings. Diuresis renography is a safe and widely available provocative test that, in addition to evaluating the renal function, allows evaluation of the extent and pattern of clearance of the radiopharmaceutical from the dilated urinary tract under high diuresis (1,2). The results of diuresis renography are dependent on several physiologic, anatomic, mechanical, and technical factors, including function of the affected kidney, capacity and compliance of the dilated system, the patient’s hydration status, fullness of the bladder, dose of furosemide, time of injection of furosemide relative to injection of the radiopharmaceutical, and selection of regions of interest for generation of time–activity curves. Understanding the principles of the test, its limitations, and the sources of error is essential in the interpretation of the results and effective use of the test.

## II. GOALS

The purpose of this guideline is to assist nuclear medicine practitioners in performing, interpreting, and reporting the results of diuresis renography in a manner that is effective in the detection of the kidneys at risk for loss of function and helps the referring physician in the patient’s management.

## III. DEFINITIONS

1. Hydronephrosis (HN) and hydroureteronephrosis (HUN): These terms denote dilatation of the pelvicaliceal system or pelvicalyceal system and ureter, respectively, that may or may not be due to obstruction.
2. Ureteropelvic junction (UPJ) obstruction: Usually refers to partial obstruction at the uretero-pelvic junction due to intrinsic narrowing or extrinsic compression by a crossing vessel. Complete obstruction of the proximal ureter during fetal development leads to nonfunctional kidney.
3. Ureterovesical junction (UVJ) obstruction: Usually refers to partial obstruction and is due to narrowing of the distal ureter, duplication with ectopic ureterocele, or ectopic insertion of the ureter.
4. Acute UPJ obstruction: This condition usually occurs in older children, manifested by sudden onset of severe flank/abdominal pain associated with nausea/vomiting (Dietl’s crisis). These episodes are often recurrent and are usually due to intermittent obstruction without urinary calculi. Nephrolithiasis is uncommon in children, but it may cause acute urinary tract obstruction (UPJ or UVJ). Acute UPJ obstruction occasionally occurs during diuresis renography.
5. F-0 protocol: A diuresis renography technique with simultaneous administration of furosemide and radiopharmaceutical.
6. F+(20 or 30) protocol: Diuresis renography techniques with administration of furosemide 20 or 30 min after administration of radiopharmaceutical.

## IV. COMMON CLINICAL INDICATIONS

1. Initial evaluation of renal function and drainage in asymptomatic infants and children with sonographic diagnosis of HN/HUN and voiding cystourethrography demonstrating either no evidence of VUR or findings suggestive of coexistent reflux and UPJ obstruction.
2. Serial follow-up examinations in patients with an inconclusive initial study to detect changes in the renal function or progressive worsening of postdiuresis drainage in an attempt to detect the kidneys at risk for loss of function.
3. Older children with recurrent abdominal pain and HN to diagnose intermittent UPJ obstruction as the cause of the symptoms. Diuresis renography in these patients would be most informative if it is done during or within 24 h after an episode of pain (3).
4. Evaluation of renal function and postdiuresis drainage after surgical interventions.

## V. QUALIFICATIONS AND RESPONSIBILITIES OF PERSONNEL

In the United States, see Section V of the SNMMI Guideline for General Imaging. In Europe, the certified nuclear medicine physicians who perform the study and sign the report are responsible for the procedure, according to national laws and rules.

## VI. PROCEDURES/SPECIFICATIONS OF THE EXAMINATION

### A. Patient preparations and precautions

1. **Parent information**  The parents should be given detailed information about the examination at the time of scheduling. Preparation before arrival in the department is usually not necessary if the procedure protocol includes routine administration of intravenous hydration. Otherwise, oral hydration before arrival and while in the department should be encouraged. Fasting is unnecessary and should be avoided. In rare cases, when the study will be done under sedation, the guidelines of the institution must be followed and the patient must be hydrated during examination by intravenous administration of fluid (see below).
2. **Before the examination**  The supervising nuclear medicine physician should review all available clinical, laboratory, and imaging data. In particular, knowledge of relevant urologic procedures and surgeries such as presence of nephrostomy tube, ureteral stent, and urinary diversion is essential in the technical planning of the test and interpretation of the results. The nephrostomy tube should be clamped before examination.
3. **Patient education**  The procedure should be explained to the parents and children old enough to understand. The parents should be encouraged to stay with the child throughout the examination.
4. **Anesthetic cream**  Application of an anesthetic cream or spray on the potential venous access site(s) is advised.
5. **Intravenous access**  With the F-0 technique, the tracer and furosemide can be injected through a butterfly needle of appropriate size. With the F+(20 or 30 min) technique, however, it is necessary to establish a secure intravenous access using an intracath to be used for initial injection of the tracer and subsequent injection of furosemide in a timely fashion (see D-5), as well as infusion of intravenous fluid per protocol.
6. **Hydration**  Adequate hydration before or during examination is essential to achieve optimal postfurosemide diuresis and prevent dehydration. This is particularly important in infants and young children. Intravenous hydration with 5% dextrose in 0.33 normal saline or any other solution per the institution’s policy is essential in patients who cannot or will not comply with oral intake of fluids. The suggested volume of intravenous fluid is 15–20 mL/kg (about two thirds of it should be given before furosemide injection). However, many children can achieve adequate hydration by age-appropriate oral intake of fluids (milk, water, juice) when clear instructions are presented to them (or to their caregivers) and when compliance with these instructions can be monitored.
7. **Eliminating/minimizing potential effects of a full bladder**  A full bladder may result in significant increase in intravesical pressure and cause delayed drainage from the renal pelvis. In toilet-trained children, a full bladder may also cause premature interruption of the study due to the need of the patient to void. Therefore, it is important to eliminate or minimize overdistention of the bladder during the examination. This could be achieved by insertion of an indwelling bladder catheter allowed to drain freely into a closed collection bag. An additional advantage of continuous drainage of the radioactive urine from the bladder is significant reduction in gonadal radiation dose. However, catheterization, for the child, is an invasive and unpleasant procedure. Therefore, its routine use is controversial. But it is advised in infants and children with HUN, PUV, known VUR, or neuropathic bladder.

 In the absence of an indwelling bladder catheter, all toilet-trained patients should be asked to empty their bladder before acquisition of the dynamic images. All boys who are able and willing to void while supine, using a urinal without moving, should be asked to void a few times during the dynamic acquisition of data. All gravity-assisted drainage images should also be obtained after the patient voids (see D-6).

### B. Radiopharmaceuticals

The preferred radiopharmaceuticals for diuresis renography are ^99m^T-labeled tubular agents, because they are much more efficiently extracted by the kidney than the glomerular agents. This is particularly important in neonates and in children with severely dilated collecting systems or impaired renal function.

1. **^99m^Tc-MAG3 (tubular secretion)**

  ^99m^Tc-mercaptoacetyltriglycine (MAG3) is highly protein-bound and is removed from the plasma primarily by the proximal renal tubules (4). The extraction fraction of ^99m^Tc-MAG3 is 40%–50%, more than twice that of ^99m^Tc-diethylenetriaminepentaacetic acid (DTPA) (see below). A small fraction of the administered activity of ^99m^ Tc-MAG3 is excreted via the hepatobiliary system. Therefore, visualization of the tracer in the gallbladder or small bowel on the delayed images is a normal physiologic finding and should not be mistaken for retention in the right renal pelvis or urine leak.

2. **^99m^Tc-EC (tubular secretion)**

  ^99m^Tc-l,l- and -d,d-ethylenedicysteine (EC) are enantiomers. Both are excellent renal radiopharmaceuticals with clearances slightly higher than ^99m^Tc-MAG3 (5). Although ^99m^Tc-d,d-EC is cleared more rapidly than ^99m^Tc-l,l-EC, ^99m^Tc-l,l-EC was first described, and it is available as a kit formulation in several countries but not in the United States.

3. **^99m^Tc-DTPA (glomerular filtration)**

  ^99m^Tc-DTPA is purely filtered by the glomeruli (6). Although its extraction fraction is approximately 20%, it can be used for diuresis renography if tubular agents are unavailable.

### C. Equipment specifications

A large field-of-view γ-camera equipped with a low-energy high-resolution collimator is recommended but a low-energy all-purpose collimator is an acceptable alternative. A 128 × 128 matrix with zoom (magnification) set to produce a pixel size of less than 4 mm should be used. γ-camera quality control should follow national rules or manufacturer’s instructions. For further guidance on routine quality control procedures for γ-cameras, refer to the SNMMI Guideline for General Imaging and the EANM guideline on routine quality control for nuclear medicine instrumentation.

### D. Image acquisition

1. **Patient position**
   The images are acquired with the patient supine on the imaging table.
2. **Camera position**
   The camera is usually under the table for acquisition of dynamic and static posterior images of normally positioned kidneys. If the affected kidney is a horseshoe variant or in an ectopic location, the posterior images alone would not be adequate for accurate calculation of the differential renal function (DRF). In this situation, if it is feasible, the initial images should be acquired with a dual-detector camera for geometric mean calculation of the DRF, but posterior images are adequate for evaluation of the postdiuresis drainage. The images for evaluation of HN in transplanted kidneys must be acquired anteriorly.
3. **Administered activity**
   All administered activities in children should be scaled down according to the tables provided by either the EANM or the SNMMI (**7**).
4. **Dose of furosemide**
   The standard dose of furosemide is 1 mg/kg with a suggested maximum dose of 40 mg.
5. **Image acquisition protocols**
   Several protocols have been used for diuresis renography based on the administration of furosemide before, simultaneous with, or at varying intervals after administration of the radiopharmaceutical (8,9). The most commonly used protocols are F-0 and F+(20 or 30 min).
   In the F-0 protocol, furosemide is administered simultaneously with the radiopharmaceutical, and dynamic posterior images (10–15 s per image) are obtained for 20 min (10).
   In the F+20 or F+30 protocols, the radiopharmaceutical is injected intravenously, and dynamic posterior images (10–15-s per image) are acquired for 20 or 30 min, followed by intravenous administration of furosemide and acquisition of a second set of dynamic images (10–15 s per image) for 20 or 30 min. However, furosemide should ideally be injected after the entire dilated collecting system is filled with radioactive urine and there is adequate residual tracer activity for a meaningful evaluation of postdiuresis drainage parameters. Therefore, in the following situations it may be necessary to modify the timing of furosemide administration:
  a. The dynamic images should be reviewed immediately after acquisition. In severely dilated systems particularly with dilated ureter, the entire system may not be filled with radioactive urine at the end of 20 or 30 min of dynamic imaging. These patients should be kept upright or prone for a few minutes to achieve better filling of the entire dilated system and more homogeneous distribution of the radiotracer before administration of furosemide.
  b. If monitoring of the initial dynamic images during acquisition shows fast drainage of the tracer from the dilated collecting system that may result in inadequate residual activity at the end of 20 or 30 min of acquisition, furosemide should be administered earlier to evaluate drainage under high diuresis.
6. **Gravity-assisted and postvoid drainage**
   Regardless of the protocol used, the quality of drainage must be assessed both visually and semiquantitatively at the end of the postfurosemide dynamic imaging because drainage could be affected by the supine position of the patient and, in the absence of indwelling bladder catheter, by varying degrees of bladder filling. Therefore, if drainage at the end of dynamic imaging is insufficient (high residual activity), it must be reassessed after the patient is kept upright for a standardized period of time and, in the absence of indwelling bladder catheter, after he/she empties his/her bladder (11). The following imaging techniques are commonly used for evaluation of gravity-assisted/postvoid drainage:
  a. Static 1-min posterior images are obtained before and after the patient is kept upright for a standardized period of time (e.g., 10 or 15 min).
  b. Static 1-min image is obtained at a standardized time period (60 min or longer) after the radiopharmaceutical injection.

### E. Data processing

1. **Calculation of DRF**
    Calculation of the DRF should be based on the summed image with the maximum accumulation of the radiopharmaceutical in the parenchyma and no radiopharmaceutical in the pelvicalyceal system, usually the second minute (60–120 s) images. The renal region of interest (ROI) should include only the entire functioning renal tissue. The preferred ROI for background subtraction is, around and slightly separated from the renal ROI, excluding the hilum of the kidney. This C-shaped ROI appropriately includes the contributing counts from the liver and spleen blood-pool activity. Background-subtracted counts in each kidney are used for calculation of the DRF by using the integral method (area under the curve) or the Rutland-Patlak plot (12–15).
2. **Calculation of drainage parameters**
  a. Generation of time–activity curves. Postfurosemide dynamic images are used for evaluation of the pattern and speed of clearance of the radiotracer from the dilated pelvicalyceal system and ureter. Attention must be paid to the following crucial points in the processing of the postdiuresis data and generation of the time–activity curves:
    - Motion correction. The dynamic images must be displayed in a cinematic form to detect and correct for patient movement during the acquisition time.
    - The ROI for generation of the postdiuresis time–activity curve must include the entire dilated system. In the case of HN, it should include the entire dilated pelvicalyceal system. But if the ureter is also dilated, it must be included in the ROI (single ROI). However, in a small percentage of cases of HUN, the level of obstruction, if any, may not be clear. For example, if the pelvicalyceal system is disproportionately more dilated than the ureter, it is important to exclude UPJ obstruction by adding a separate ROI for the pelvicalyceal system (double ROIs) or even a third ROI for dilated ureter (triple ROIs).
    - The images with maximal dilatation of the collecting system must be chosen for drawing the ROI for generation of the postdiuresis time–activity curve. After injection of furosemide, the dilated renal pelvis may gradually become more dilated and if the early postfurosemide images are chosen for drawing the ROI, the entire dilated pelvis will not be included in the ROI on all images and the resultant time–activity curve would not be true representative of postdiuresis clearance of the tracer from the dilated pelvis.
  Processing of the time–activity curves. Basic semiquantitative indices derived from the postdiuresis drainage time–activity curve, using commercially available softwares, are the washout half-time (T1/2) and the percentage drainage at the end of 20- or 30-min \dynamic acquisition. A more robust measurement of postdiuresis drainage is expressing residual counts in the renal parenchyma and the dilated pelvicalyceal system/ureter over a period of 1 min at the end of dynamic imaging as percentage of the renal counts in the second minute after injection of the radiopharmaceutical (normalized residual activity [NORA]) (16,17).
  Evaluation of gravity-assisted/postvoid drainage. With the technique using static pre- and postupright images obtained shortly after completion of dynamic imaging (see D-6a), the gravity-assisted drainage is simply calculated by using the total counts in the ROIs drawn around the entire dilated system on both images and using the following formula (background activity and decay factor are negligible):
    - Gravity-assisted drainage = (counts in the preupright image ROI) minus (counts in the postupright image ROI) divided by (counts in the preupright image ROI).
    - With the technique using a single delayed image (see D-6b), the gravity-assisted/postvoid drainage is assessed either visually or by NORA.

### F. Postprocessing image display

1. The dynamic pre- and postfurosemide images should be reformatted and displayed as 1- or 2-min images for visual evaluation of the kidney morphology, cortical clearance, and postdiuresis drainage as well as associated anomalies such as duplication.
2. The image demonstrating the DRF should include the time–activity curve of dynamic images, the summed image of the kidneys, and the ROIs used for calculation of the DRF.
3. The postdiuresis drainage result should include the summed image, the ROIs, and the drainage time–activity curve. Depending on the protocol used, all semiquantitative indices should be included.
4. The gravity-assisted drainage data should include the pre- and postupright static images with the ROIs and calculated percentage drainage or NORA depending on the acquisition technique.

### G. Interpretation

The interpreting physician should carefully review all the images, the shape of the drainage curve (drainage pattern), the quality of cortical clearance of the tracer, and all available semiquantitative indices (DRF, postfurosemide drainage T1/2, percentage drainage at the end of dynamic imaging, gravity-assisted drainage, or NORA). The scintigraphic findings and semiquantitative drainage parameters are interrelated, and none of them should be interpreted in isolation. This is particularly true for T1/2. Interpretation of the result must be based on the constellation of the findings and in the clinical context (18–20).

On any single study, diagnosis of high likelihood for critical partial obstruction and eventual loss of function can be made only if there is a flat or rising drainage curve with little or no clearance in the upright position, particularly if there is unilateral delayed cortical clearance of the tracer throughout the study or initial good clearance but with reaccumulation of the tracer in the renal cortex after furosemide administration (21). Otherwise, as most cases, the initial study will serve as a baseline for detection of a significant drop in DRF or worsening of postdiuresis drainage on the follow-up examinations. Therefore, it is important to adopt the same protocol and data processing techniques for the baseline and all follow-up studies in each patient. True and significant decrease in DRF between 2 studies in a patient is usually associated with a relative delay in appearance of the tracer in the renal calyces (prolonged cortical transit time) (11).

## VII. DOCUMENTATION/REPORTING

The report should include patient demographics, clinical indication for the study, prior surgical interventions, and the pertinent results of other imaging studies. If hydration is administered in the department, its volume and route of administration should be recorded. The volume of total urine output during the examination, if known, should be reported. The presence of a nephrostomy tube with the information about the time interval that it was clamped during the study must be reported. The administered activity and the dosage and timing of furosemide during the procedure must be clearly cited.

The size, shape, and position of the kidneys and any focal defect in cortical uptake should be described. Locations of noteworthy tracer retention in parenchyma, calyces, renal pelvis, and ureters on pre- and/or postfurosemide images should be noted.

The DRF and all calculated drainage parameters including the basic indices such as T1/2 and the percentage drainage at the end of dynamic imaging as well as any additional quantitative measurements of drainage such as gravity-assisted drainage and NORA should be reported. The shape of the abnormal postfurosemide time–activity curves (e.g., flat, rising, biphasic) should be described.

The absence or presence and location of pain during examination must be documented because the presence of diuretic-induced flank pain may increase the likelihood that obstruction is present. Inclusion of an impression/conclusion at the end of the report is recommended.

Inclusion of a statement about radiation burden should follow national rules.

## VIII. QUALITY CONTROL AND SOURCES OF ERROR

Quality control issues and potential pitfalls have been reviewed in previous sections.

## IX. SAFETY, INFECTION CONTROL, AND PATIENT EDUCATION CONCERNS

See the SNMMI Guideline for General Imaging.

## X. RADIATION DOSIMETRY

See International Commission on Radiological Protection publications 23, 26, and 38 (22–24) and also Stabin and Gelfand (25).

## XII. APPROVAL

This standard/guideline (version 2.1) was approved by the Board of Directors of the SNMMI on June 22, 2018.
